# Neglecting the Left Side of a City Square but Not the Left Side of Its Clock: Prevalence and Characteristics of Representational Neglect

**DOI:** 10.1371/journal.pone.0067390

**Published:** 2013-07-10

**Authors:** Cecilia Guariglia, Liana Palermo, Laura Piccardi, Giuseppe Iaria, Chiara Incoccia

**Affiliations:** 1 Dipartimento di Psicologia, Sapienza Università di Roma, Rome, Italy; 2 Lab. Di.Vi.Na., I.R.C.C.S. Fondazione Santa Lucia, Rome, Italy; 3 Dipartimento di Medicina Clinica, Sanità Pubblica, Scienze della Vita e dell'Ambiente, Università degli Studi di L'Aquila, Coppito (AQ), Italy; 4 NeuroLab, Departments of Psychology and Clinical Neurosciences and Hotchkiss Brain Institute, University of Calgary, Calgary, Alberta, Canada; Tokyo Metropolitan Institute of Medical Science, Japan

## Abstract

Representational neglect, which is characterized by the failure to report left-sided details of a mental image from memory, can occur after a right hemisphere lesion. In this study, we set out to verify the hypothesis that two distinct forms of representational neglect exist, one involving object representation and the other environmental representation. As representational neglect is considered rare, we also evaluated the prevalence and frequency of its association with perceptual neglect. We submitted a group of 96 unselected, consecutive, chronic, right brain-damaged patients to an extensive neuropsychological evaluation that included two representational neglect tests: the Familiar Square Description Test and the O'Clock Test. Representational neglect, as well as perceptual neglect, was present in about one-third of the sample. Most patients neglected the left side of imagined familiar squares but not the left side of imagined clocks. The present data show that representational neglect is not a rare disorder and also support the hypothesis that two different types of mental representations (i.e. topological and non-topological images) may be selectively damaged in representational neglect.

## Introduction

Hemineglect (or neglect) is a pervasive disorder of space representation that occurs following lesions of posterior areas of the right hemisphere [1]. It is characterized by the inability to orient attention or consider events in the contralesional side of extrapersonal or personal/body space. Patients affected by neglect lose the ability to react to or process sensory stimuli (visual, auditory, tactile, olfactory) presented in the contralesional hemispace. “Sensory neglect” is also referred to as perceptual neglect [2]. Finally, exploratory-motor neglect may manifest as reduced use or non-use of a contralateral body part (i.e. arm, leg) during walking and daily life activities [3].

As hemineglect is a very complex disorder, which can affect several components of spatial behaviour in different ways, two tests tapping different aspects of hemineglect may produce contrasting results and show different degrees of neglect in the same patient [4]. Consequently, formal testing has to be exhaustive and include tasks that assess different aspects of hemineglect. Several dissociations have been described in patients with neglect that clearly demonstrate the complexity of the neglect syndrome [5] [6]. For example, dissociations between extrapersonal and personal space [7] [8] have been described as well as dissociations between perceptual and imaginary space [9]–[12]. When difficulty in conjuring up the left side of mental images is present, it is referred to as representational or imagery neglect. Representational neglect is the inability to process the contralesional side of visual mental images. Bisiach and Luzzatti [13] first described the disorder in two right brain-damaged patients with visuo-spatial neglect. Since publication of their seminal paper, selective impairment in describing the left side of familiar places from memory or in processing the left side of mental images of real or abstract objects has been considered rare in patients with neglect [14] [15].

There has always been a certain theoretical interest in representational neglect and several hypotheses have been proposed to explain its nature. First, representational neglect is considered analogous to perceptual neglect, that is, the same exploration bias that affects visual perception in neglect impedes the exploration of the contralesional side of visual mental images [16]. Although representational neglect is usually found in patients also affected by perceptual neglect, the existence of dissociated cases of representational neglect in the absence of perceptual neglect undermines this interpretation [9] [12] [17] [18].

A deficit in directing attention toward the left side of mental images or damage to the mental representation system, that is, a “tearing” of the left side of the mental screen, has also been hypothesized [16]. Logie and co-workers [19] suggested a deficit in generating the left side of mental images in a study in which two patients affected by representational neglect had to describe an array of four objects from memory. The patients were shown one out of ten different arrays. After the array was removed from sight, they had to describe it from memory in the same perspective they had seen it in before or from the opposite (180° of rotation) perspective. Both patients consistently failed to report objects on the left side of their mental images but showed no defect in reporting objects on the right side; that is, after mental rotation they were able to describe items they perceived on the left side of the array when these objects were located on the right side of the mental image.

Other authors have suggested that representational neglect results from a) damage to a system involved in the generation of mental images that is independent from systems involved in exploring and representing visuo-spatial percepts [9]; b) damage to an egocentric spatial representation involved in the maintenance of visual information across saccades and time [17]; and c) unilateral damage to visuo-spatial working memory [18].

All of these interpretations consider representational neglect as a defect involving all types of visual mental images. Nevertheless, cases of dissociations in representing different types of mental images have been reported. Grossi et al.'s patient [20] was severely impaired in judging the left side of mental images of real objects on the O'Clock Test but showed no asymmetry in describing a familiar public square from memory. Guariglia et al.'s patient [9], who showed selective representational neglect in describing familiar squares from memory, showed no asymmetry in processing the left side of mental images of objects. Furthermore, Ortigue and co-workers [21] recently reported a patient who was unable to visualize the left side of mental images of landscapes and maps but was perfectly able to visualize the left side of the interior of her car or describe the left side of visual arrays of objects from memory. Very recently, Arduino et al. [22] reported a dissociation in mentally inspecting words or objects in patients suffering from neglect. This finding suggests that a specific mechanism exists for processing orthographic material with respect to other types of stimuli. Therefore, it can be hypothesized that different systems process different types of mental images. Classical models of mental imagery distinguish between two different types of visual mental images: skeletal images, which have few details and low resolution, and complex images, which are multipart, high-resolution images [23]. This distinction does not help explain the dissociations in representational neglect, because most of the tasks used to test representational neglect for objects require generating complex mental images (e.g., the O'Clock Test [20], the slit tests [24] [25], or descriptions of visual arrays from memory [21] [19]). Some recent findings suggest a different distinction based on different types of visual mental images, namely, topological and non-topological images [26]. Topological images are mental representations of stimuli in which the subject can navigate (i.e., rooms, squares, cities, maps, etc.) and that can be transformed into (or correspond to) cognitive maps of the environment. Non-topological images are mental representations of stimuli, such as a desktop, the interior of a car [21], single objects or arrays of objects, which can be manipulated but never navigated. Although topological and non-topological mental images may have some mechanisms in common, the above-described dissociations suggest that they are essentially processed by different systems. Two different types of data support this hypothesis. The first type comes from recent findings that patients with representational neglect are significantly impaired in navigational tasks based on mental representations (cognitive maps) of the environment [27]–[30]. Indeed, Palermo et al. [29] reported a right brain-damaged patient who showed selective representational neglect in tasks requiring the description of familiar environments from memory but no representational neglect in tasks requiring mental representation of single and multiple objects. Interestingly, this patient also showed consistent navigational deficits but no sign of perceptual neglect. The link between topological mental images and navigation skills is supported by Palermo and colleagues' recent findings [31]. These authors reported that representational neglect patients showed selective mental imagery deficits on tasks involving the generation, inspection and transformation of mental images of environments.

Another type of evidence comes from a second reading of previously described cases and suggests differences in the processing of topological and non-topological images. In fact, in Grossi et al.'s patient [20] and in the patients described by Guariglia et al. [9] and Ortigue et al. [21] dissociated performance on representational tasks can be interpreted as selective impairments in processing non-topological [20] or topological [9] [21] mental images.

In the present study, we evaluated a large group of right brain-damaged patients to test the hypothesis of two distinct forms of representational neglect, one involving the representation of topological images and the other, the representation of non-topological images. As representational neglect is considered rare, its prevalence and frequency of association with perceptual neglect was also evaluated in a group of unselected, consecutive, chronic patients.

## Methods

### Participants

We recruited right brain-damaged patients consecutively admitted to the I.R.C.C.S. Fondazione Santa Lucia in Rome who showed no comprehension deficits or mental decay on the psychological examination at admission. The study protocol was approved by the local ethics committee (IRCCS Fondazione Santa Lucia of Rome, Italy) following the ethical principles in the Declaration of Helsinki. All patients were compos mentis and signed written consent forms before taking part in the experimental testing. The examiners explained the purpose of the research to the patients and responded to their questions and concerns. Exclusion criteria included a history of multiple cerebrovascular accidents, general cognitive decay, previous neurological or psychiatric disorders and an uncertain diagnosis of perceptual neglect (one patient performed below the cut-off on only one of the four screening tests; see below).

The study included 96 patients: 25 (26.04%) females (F) and 71 males (M) (73.96%); mean age was 63.88 years (S.D. = 11.01 years), mean years of education, 9.39 years (S.D. = 4.65 years), and mean distance from onset, 381.28 days (S.D. = 831.41 days).

A control group of 30 healthy participants matched for age, gender and education with the right brain-damaged patient group (age: mean = 66.09 years, S.D. = 9.36 years; education: mean = 9.33 years, S.D. = 3.60 years) was also recruited to obtain the normal degree of asymmetries on the visual imagery tests used to evaluate representational neglect. An ANOVA showed that patients did not differ from healthy participants for age (F_1,120_ = 0.80; n.s.) or education (F_1,120_ = 0.32; n.s.). Each healthy participant was submitted to the MMSE [32] to rule out the presence of general, as yet unrecognized, mental deterioration.

### Testing

All patients were submitted to an extensive neuropsychological examination to assess their orientation in time and space, personal orientation [33], language functions [34], visuo-spatial and verbal short-term and working memory [33], long-term verbal memory [33], abstract and/or verbal reasoning [35] [33], attention, constructional apraxia, visuo-perceptual skills [33] [36] and visual agnosia [33]. Patients' performance on the neuropsychological examination was used to exclude the presence of general mental decay and visuo-spatial disorders not restricted to the contralesional hemifield.

A standard battery for evaluating the neglect syndrome [37] was used to determine whether perceptual neglect was present and, if so, its severity. The battery includes four conventional tests:

Letter Cancellation Test [38]: Subjects' task is to cross out 104 uppercase “H's” interspersed among 386 different letters arranged in 6 horizontal lines on a sheet of A3 paper (total score range 0–104; 0–53 on the left, 0–51 on the right). The sheet is presented centrally in front of the patient. The cut-off is a difference of ≥4 between omissions on the left and on the right side. The maximum number of omission errors in healthy subjects is four; the maximum difference between errors on the left and the right is two [39].

Line Cancellation Test [40]: 21 lines with different orientations (3 cm long) are randomly dispersed on a sheet of A3 paper presented centrally in front of subjects (total score range 0–21; 0–11 on the left, 0–10 on the right). They have to cross out all the lines they can find without a time limit. The cut-off is ≥2 omissions on the left side. Healthy subjects make no errors on this test.

Wundt-Jastrow Area Illusion Test [41]: Subjects are presented with a picture of two identical black fans placed one above the other so that one of them appears horizontal; they have to point to the stimulus that seems longest (illusionary effect). In 20 trials, the illusory effect is present in left-oriented and in 20 trials in right-oriented stimuli. In neglect patients, the illusory effect is reduced on the contralesional side [41]. The score is the number of trials in which the normal illusory effect is present on each side (score range 0–20). The cut-off is the difference >2 between unexpected responses (i.e., responses in the direction opposite the illusory effect in controls) for left-oriented minus right-oriented stimuli.

Sentence Reading [37]: The patient has to read aloud six sentences ranging from 5 to 11 words (21–42 letters). The score is the number of correctly read sentences (score range 0–6). The cut-off is one or more sentences read incompletely on the left side. Healthy subjects and right brain-damaged patients without hemineglect make no errors. Patients with neglect (as reported in the original paper by Pizzamiglio and co-workers) [37] make omission errors, substitution errors, or both in the left half of the sentence.

In accordance with normative rules, the patients were classified as affected by perceptual neglect (PercNeg) if they scored below the cut-off on at least two of the four tests.

The Standardized Battery was adopted because it is the diagnostic instrument most used to evaluate neglect in Italy and includes a standardized reading test for the Italian population. Furthermore, the tests included in this Battery allow assessing different aspects of neglect. Specifically, at variance with the Line Cancellation and Letter Cancellation tests, the Wundt-Jastrow Area Illusion Test and the Sentence Reading Test have the advantage of not requiring a limb-motor response and thus allow testing visuo-perceptual components of hemineglect with minimal influence of motor components (both tests involve only ocular movements).

Two tests were used to assess representational neglect (ReprNeg): the Familiar Square Description Test (derived from Bisiach and Luzzatti) [13] to assess whether ReprNeg for topological images was present, and the O'Clock Test or the Mental Clock task [42] [20] to assess whether ReprNeg for non-topological images was present. Although a dissociation between full representation of visual events (objects, faces, written material, etc.) and defective representation of the left side of the environment has already been described [9] [21], no clinical studies have systematically investigated the presence of these two different types of mental representation. To investigate this issue, we used the Familiar Square Description Test and the O'Clock Test. The former allows studying the mental representation of an environment that was well-known before illness onset and whose mental map was developed by direct navigational experience; the latter allows investigating the mental representation of a well-known familiar object whose mental image is not related to a motor-proprioceptive action but only to perceptual knowledge.

In the Familiar Square Description Test, patients had to describe two familiar public squares from memory from two opposite vantage points. The elements described on each side of the square were recorded. When the patient described a square unknown to the experimenters, a relative was asked to describe the square in detail before the patient was tested. The relative's description was used to choose the two vantage points and to score the patient's performance. The number of elements reported on the left and the right side of the imagined squares was transformed into a laterality quotient as follows: LQ = (left elements-right elements/left elements+right elements) *100 [14]. An LQ with a negative sign meant that the subject reported fewer elements on the left than the right side; vice versa, a positive sign indicated the opposite behaviour. In the control group, the mean LQ was −1.46 (S.D. = 7.39) and ranged from −15.28 to 11.11; to avoid false positive errors, we decided to adopt an LQ equal or higher than −20 as the cut-off for the presence of ReprNeg for topological images because this score was higher than the score of the worst control performance. Therefore, participants were classified as affected by ReprNeg for topological images (T-ReprNeg) if their LQ was equal or lower than −20.

The O'Clock Test [20] requires the generation of multipart mental images [43]. Patients were asked to imagine two different times on two analogical clocks and to decide which clock hands formed the widest angle. We used 32 time pairs. They included only half hours (e.g., 7:30) or hours (e.g., 9:00), balanced for correspondence with numerically greater or smaller times (e.g., 3:00 >1:00) and for the visual hemifield that corresponded with the position of the imagined clock hands (e.g., 16 pairs of times were in the right hemifield, that is, 3:00 and 5:30, and 16 were in the left hemifield, that is, 9:00 and 7:00). Scores were the number of correct responses. Before testing, a training session (8 trials) was carried out in which a perceptual version of the test was proposed. During training, two analogical clocks were shown in a vertical array, but the pairs of times were different from those used in the test (e.g., 3:20 and 5:05; 7:50 and 10:45). Also for the O'Clock Test, we adopted the same above-mentioned formula for calculating a Laterality Quotient [14]: LQ = (correct responses on the left−correct responses on the right/correct responses on the left+correct responses on the right)*100. Negative LQ refers to fewer correct responses on the left. In the control group, the mean LQ was −1.10 (S.D. = 6.41) and ranged from −16.67 to 15.79. To avoid false positive errors, we decided to adopt an LQ cut-off of −20, which was lower than the worst control score. Therefore, patients were classified as having representational neglect for non-topological images (Nt-ReprNeg) if their LQ was equal to or lower than −20.

Correlation between the Familiar Square Description Test and the O'Clock Test was computed in the control group (r = 0.16).

## Results

**Figure 1:**
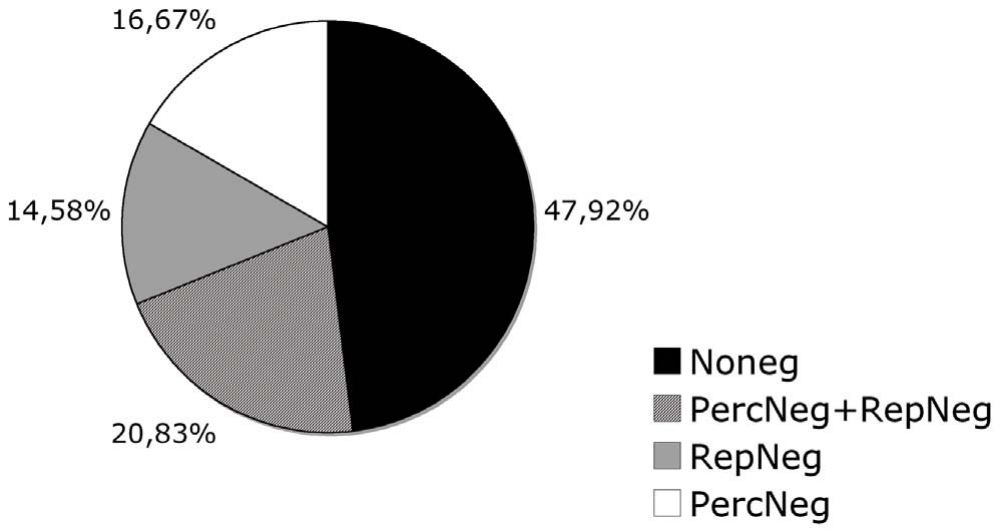
This figure reports the percentage of patients with perceptual neglect (PercNeg), representational neglect (RepNeg), perceptual neglect associated with representational neglect (PercNeg+RepNeg) and no signs of neglect (Noneg).

First, we determined the frequency of occurrence of perceptual and representational neglect in a sample of 96 right brain-damaged patients (see Fig. 1). We found that 50 patients had some form of neglect: 36 patients had perceptual neglect with or without signs of representational neglect (37.50% of the total sample) and 14 showed pure representational neglect without signs of perceptual neglect (RepNeg: 14.58% of the total sample). Regarding the 36 patients with perceptual neglect, 16 (PercNeg: 16.67% of the total sample) showed no signs of representational neglect and 20 (PercNeg+RepNeg: 20.83% of the total sample) were also affected by representational neglect (see Fig. 1).

**Figure 2:**
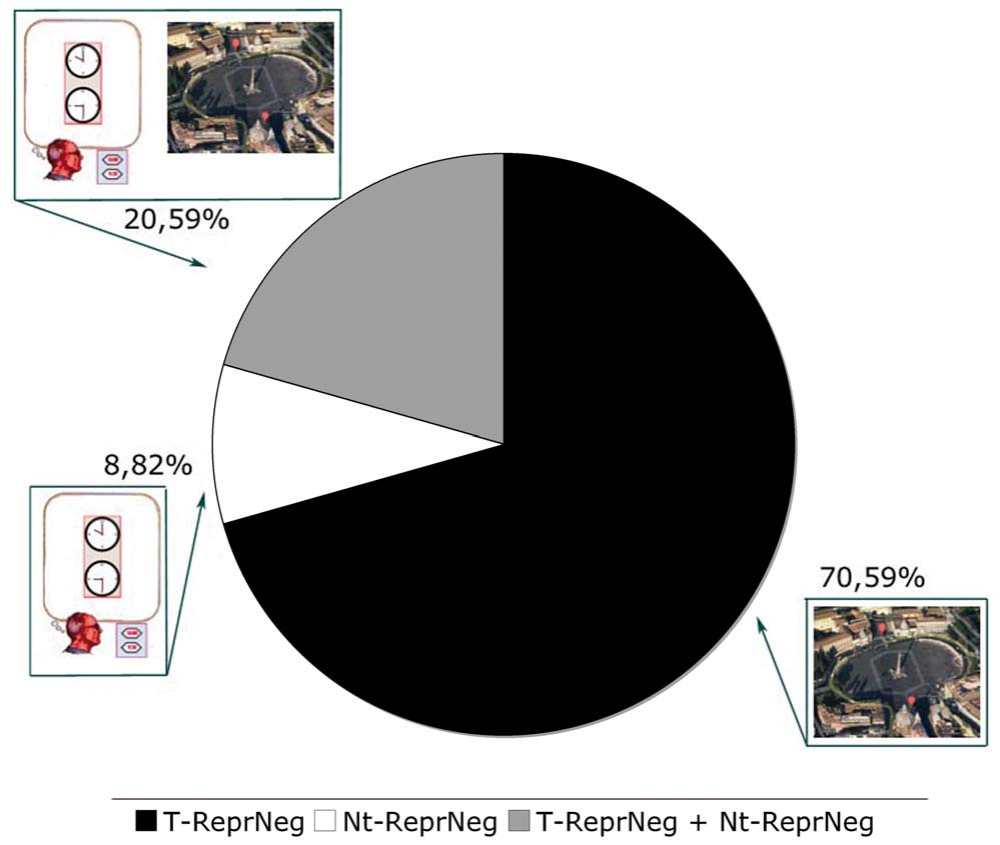
This figure reports the percentage of patients affected by selective representational neglect for topological images (T-ReprNeg), selective representational neglect for non-topological images (Nt-ReprNeg), and representational neglect for both topological and non-topological images (T-RepNeg+Nt-ReprNeg) calculated on the sub-sample of 34 patients affected by representational neglect.

Most of the 34 patients affected by representational neglect, that is, both pure (14 patients) and non pure (20 patients), showed a selective form of representational neglect for topological and non-topological images; specifically, 24 (T-RepNeg: 25% of the total sample) showed selective representational neglect only in the Familiar Square Description Test, 3 (Nt-RepNeg: 3.13% of the total sample) showed selective representational neglect only in the O'Clock Test, and the remaining 7 patients (7.29% of the total sample) showed both T-ReprNeg and Nt-ReprNeg (see Figure 2).

The frequency of the different forms of neglect was roughly similar in right brain-damaged patients with PercNeg (14.58%), RepNeg (16.67%), and PercNeg+RepNeg (20.83%), but the difference was not significant (Chi-square = 1.12; df = 2; n.s.). By contrast, the frequency of the various forms of representational neglect was different (Chi-square = 21.95; df = 2; p<.01) in T-RepNeg (25%), Nt-RepNeg (3.31%) and T-RepNeg+Nt-RepNeg (7.29%).

Means and SD of the two representational neglect tests are reported in Table 1.

**Table 1:** Means and (SD) of LQ obtained in Familiar Square Description Test (T-LQ) and O'Clock Test (Nt-LQ).

Groups	T-LQ	Nt-LQ
RepNeg (n. 14)	−32.25 (15.37)	−8.00 (14.43)
PercNeg (n. 16)	−0.54 (9.27)	−0.10 (11.65)
PercNeg+RepNeg (n. 20)	−33.80 (23.68)	−6.76 (7.39)
Noneg (n. 46)	1.59 (12.19)	−1.64 (8.54)
Controls (n. 30)	−0.96 (6.01)	−1.26 (7.35)

To assess differences among groups in the representational neglect tests, we performed a MANOVA with Group (right brain-damaged patients and controls) as independent variable and the LQ in the Familiar Square Description Test and the LQ in the O'Clock Test as dependent variables.

A significant difference among Groups (Wilks Lambda _2,120_ = 0.948; p<.05) was revealed. A post-hoc test (Duncan test) demonstrated that patients differed from controls in the Familiar Square Description Test (p<.05) but not in the O'Clock Test (n.s.). This finding suggests either that RepNeg is more frequent if assessed with the Familiar Square Description Test or that topological image representational neglect is more frequent than non-topological image representational neglect.

Pearson correlations were also calculated to verify whether the two representational neglect tests (Familiar Square Description Test; O'Clock Test) correlated in a different way with the Standard Battery Tests. Namely, we verified whether performance on the Familiar Square Description Test was correlated with that on the Letter Cancellation Test and the Line Cancellation Test (both of the latter tests involve egocentric frames of reference) and whether performance on the O'Clock Test (which may involve an allocentric frame of reference) was correlated with performance on the Wundt-Jastrow Area Illusion Test and the Sentence Reading Test (which mainly involve an allocentric frame of reference). Results failed to reveal a specific relationship. Indeed, performance on the Familiar Square Description Test showed low correlations with one of the egocentric tests (Line Cancellation Test: r = 0.28, p<0.01) and one of the allocentric tests (Sentence Reading Test: r = 0.24, p<0.05), but no significant correlations with the other Standard Battery Tests (Letter Cancellation Test: r = 0.12, n.s.; Wundt-Jastrow Area Illusion Test: r = 0.16, n.s.). The O'Clock Test did not correlate with the Standard Battery of Tests involving an allocentric frame of reference (Wundt-Jastrow Area Illusion Test: r = 0.16, n.s.; Sentence Reading Test: r = −0.01, n.s.) or with those involving an egocentric frame of reference (Letter Cancellation Test: r = −0.1, n.s.; Line Cancellation Test: r = 0.15, n.s.).

Two independent factors were extracted from a factor analysis using a principal component solution and a Varimax rotation with Kaiser Normalization of the factor matrix (retaining only factors with eigenvalues greater than 1) on the four Standard Battery Tests and on the two representational neglect tests (see Table 2 for details). The first factor, with the Letter Cancellation Test, Line Cancellation Test, Wundt-Jastrow Area Illusion Test and Sentence Reading Test loading on it, accounted for 46% of the variance; we defined this as the “Visual Perception” factor. The second factor, with the Familiar Square Description Test and the O'Clock Test loading on it accounted for 18% of the variance; we defined this as the “Mental Imagery” factor.

**Table 2:** Rotated factors matrix.

	Factors	
	Visual Perception	Mental Imagery
Letter Cancellation Test	**.819**	−.044
Line Cancellation Test	**.784**	.317
Wundt-Jastrow Area Illusion Test	**.825**	.235
Sentence Reading Test	**.780**	−.009
Familiar Square Description Test	.222	**.524**
O'Clock Test	−.085	**.874**

Factors with loadings >0.4 are indicated in bold.

A series of analyses were performed with a single-case analysis method using control participants' results to detect patients' performances in which the difference between the two ReprNeg tests was greater than expected in a normal population [44] [45]. The Crawford analysis allowed detecting: 1) defective performances, that is, those with a <.05 probability of being observed in the normal population; 2) classical dissociation, that is, patients who performed significantly below the normal range on one test (i.e., the probability of a similar performance being observed in the control population was <.05) and within the normal range on the other test; 3) strong dissociation, that is, patients who performed worse than controls on both tests, but with a discrepancy between tests with a <.05 probability of being observed in the control population.

Results showed that the performances of 25 patients were significantly different from the performances of healthy controls, with a greater discrepancy between results on the T-ReprNeg and Nt-ReprNeg tests than expected in the normal population. Nevertheless, in three of these patients both LQs were above the arbitrary cut-off; therefore, we decided to exclude these patients from the analysis of dissociations. In the remaining 22 patients, both types of dissociations were present (see Table 3). Nineteen patients showed a classical dissociation: 18 performed normally on the O'Clock Test and deficiently on the Familiar Square Description Test, and one (patient 6) showed the opposite pattern of performance. Differently, three patients presented strong dissociations: two performed significantly worse on the Familiar Square Description Test and one on the O'Clock Test. Table 3 presents statistical details (z-score for T-LQ and for Nt-LQ as well as t-tests and p) and types of dissociations. Table 4 presents results obtained by the 22 patients on the Standardized Battery for Hemineglect.

**Table 3:** Results of single case analysis performed using Crawford and Garthwaite's [44] software.

Subject	Type of neglect	Gender	Age	Education (years)	Days from onset	Left elements	Right elements	T-LQ	T z-score	Left hits	Right hits	Non-T LQ	Non-T z-score	Two-tailed t-test	p	Type of dissociation	Lesion site
1	PercNeg+RepNeg	M	65	5	70	6	9	−20.00	−4.13	12	11	4.35	1.26	3.667	0.001	classic	T - O
3	PercNeg+RepNeg	F	63	5	52	6	20	−53.85	−10.72	15	16	−3.23	−0.28	7.331	>0.001	classic	F - T- P
**6**	**RepNeg**	**F**	**82**	**11**	**312**	**12**	**18**	**−20.00**	**−4.13**	**5**	**11**	**−37.50**	**−7.29**	**2.280**	**0.036**	**strong**	ic - th
9	PercNeg+RepNeg	M	64	8	750	1	6	−71.43	−14.14	9	10	−5.26	−0.70	9.290	>0.001	classic	T - P - O
12	RepNeg	M	86	5	340	2	4	−33.33	−6.72	8	10	−11.11	−1.90	3.466	0.003	strong	//
14	RepNeg	M	75	5	42	13	22	−63.64	−12.62	10	8	−9.09	−1.85	7.799	>0.001	classic	F - i
16	PercNeg+RepNeg	M	62	18	183	2	6	−50.00	−9.97	5	9	−28.57	−5.47	3.235	0.005	strong	F - T - P - bg
17	PercNeg+RepNeg	M	60	5	327	4	2	−33.33	−6.25	8	10	−11.11	−1.90	5.785	>0.001	classic	F - T- P - bg - ic
18	RepNeg	M	72	8	97	6	10	−25.00	−5.10	16	16	0.00	0.38	3.926	0.001	classic	F - T - bg - ec - ic-
19	RepNeg	F	67	19	50	6	10	−25.00	−5.10	16	16	0.00	0.38	3.926	0.001	classic	cr - ic - ln
22	PercNeg+RepNeg	M	72	5	5479	4	10	−42.86	−8.58	10	10	0.00	0.38	6.336	>0.001	classic	P - O
35	RepNeg	M	41	19	121	14	34	−41.67	−8.35	14	16	−6.67	−0.99	5.241	>0.001	classic	F - T- P - cr - ln
41	RepNeg	M	68	5	134	10	16	−23.08	−4.73	16	16	0.00	0.37	3.662	0.002	classic	ic - ec -i
68	RepNeg	F	67	5	20	8	13	−23.81	−4.87	11	10	4.76	1.35	4.448	>0.001	classic	F - T
76	RepNeg	M	58	8	43	10	26	−44.44	−8.89	10	12	−9.09	−1.48	5.271	>0.001	classic	P
84	PercNeg+RepNeg	M	60	12	68	9	21	−40.00	−8.02	7	8	−6.67	−0.99	5.016	>0.001	classic	//
85	PercNeg+RepNeg	M	71	5	41	14	25	−28.21	−5.73	12	13	−4.00	−0.44	3.790	>0.001	classic	F - T- P - bg
**86**	**PercNeg+RepNeg**	**M**	**71**	**8**	**300**	**22**	**19**	**7.32**	**1.19**	**5**	**12**	**−41.18**	**−8.04**	**6.525**	**>0.001**	**classic**	**T - P - i**
87	PercNeg+RepNeg	M	61	5	555	3	7	−40.00	−8.02	8	9	−5.88	−0.83	5.127	>0.001	classic	//
90	PercNeg+RepNeg	F	71	8	124	7	17	−41.67	−7.45	8	6	14.29	3.30	7.536	>0.001	classic	F - T - P
91	PercNeg+RepNeg	F	69	5	112	7	17	−41.67	−7.45	8	6	14.29	3.30	7.536	>0.001	classic	F - P
94	PercNeg+RepNeg	M	65	13	74	1	5	−66.67	−13.21	13	15	−7.14	−1.09	8.447	>0.001	classic	F - P - T
	Healthy Participants	15 M; 15 F	66.09 (9.36)	9.33 (3.60)	—	18.24 (7.36)	18.48 (6.70)	−1.26 (7.35)		13.55 (2.60)	13.79 (2.34)	−0.96 (6.01)				r = 0.16	

The table reports data on patients whose performance differences on the Familiar Square Description Test (LQ T) and the O'Clock Test (LQ Non-T) were significantly higher than the difference in the controls' performance. Only patients whose performance was below the cut-off in at least one of the two tests are shown. Data of the two patients who showed more severe impairment in the Non-TRep test are indicated in bold. In the last row, mean scores of healthy participants (Control group) are reported; and the correlation between the Familiar Square Test and the O'Clock Test are reported in the last square.

Legend: PercNeg = Perceptual Neglect; RepNeg = Representational Neglect; LQ T = Laterality Quotient for the test of mental representation of topological images; z-score = z-score of LQ based on control group's performance.

bg: basal ganglia; cr: corona radiata, ec: external capsule; F: frontal lobe; i: insula; ic: internal capsule; ln: lenticular nucleus; O: occipital lobe; P: parietal lobe; T: temporal lobe; th: thalamus.

//: No RM or CT exam was performed.

**Table 4:** Table reports performances of patients in Table 1 on the Standard Battery for Hemineglect Assessment [37].

Subject	Type of Neglect	Left H (hits n. 53)	Right H (hits n. 51)	Left Line (hits n. 11)	Right Line (hits n. 10)	W-J Test (unexpected responses n. 20)	Sentence Reading (hits n. 6)
1	PercNeg+RepNeg	0*	30	11	10	13*	4*
3	PercNeg+RepNeg	16*	44	8*	10	0	4*
**6**	**RepNeg**	**53**	**51**	**11**	**10**	**0**	**6**
9	PercNeg+RepNeg	45	45	11	10	7*	2*
12	RepNeg	48	49	11	10	0	6
14	RepNeg	47	49	11	10	9*	6
16	PercNeg+RepNeg	0*	36	0*	9	16*	1*
17	PercNeg+RepNeg	0*	10	3*	9	19*	6*
18	RepNeg	53	51	11	10	0	6
19	RepNeg	52	51	11	10	0	6
22	PercNeg+RepNeg	39*	51	11	10	8*	6
35	RepNeg	51	50	11	10	0	6
41	RepNeg	53	51	11	10	0	6
68	RepNeg	52	50	11	10	0	6
76	RepNeg	52	51	11	10	1	6
84	PercNeg+RepNeg	0*	39	9*	10	8*	5*
85	PercNeg+RepNeg	0*	31	8*	10	17*	0*
**86**	**PercNeg+RepNeg**	**0** *	**12**	**11**	**10**	**7** *	**6**
87	PercNeg+RepNeg	0*	21	2*	10	3*	1*
90	PercNeg+RepNeg	46*	51	11	10	0	5*
91	PercNeg+RepNeg	45*	51	10*	10	0	6
94	PercNeg+RepNeg	50	45	0*	9	17*	1

As in Table 2, the two patients who showed more severe impairment on the Non-TRep test are indicated in bold.

Legend: PercNeg = Perceptual Neglect; RepNeg = Representational Neglect; W-J Test = Wundt-Jastrow Area Illusion Test; Left H and Right H = Letter Cancellation; Left Line and Right Line = Line Cancellation

* Denotes a deficient performance. According to Pizzamiglio and coworkers [37], a deficient performance on two out of four tests of the battery should be considered due to perceptual neglect.

Finally, as previous studies reported an association between neglect and visuo-spatial working memory deficits [46] (suggesting involvement of the visuo-spatial working memory in the representational symptoms of the neglect syndrome) [47] [48], we investigated whether patients affected by representational and/or perceptual neglect showed specific working memory deficits. A perusal of performances on the Corsi Block-Tapping Test [49] [33] showed that 1 RepNeg patient (1 out of 14; 7.14%), 5 PercNeg patients (5 out of 16; 31.25%), and 11 PercNeg+RepNeg patients (11 out of 20; 55.00%) showed defective visuo-spatial working memory. These data do not allow relating working memory deficits to a specifc type of neglect but suggest that working memory deficits are more likely to be observed in patients affected by both perceptual and representational neglect.

## Discussion

These data support the hypothesis that two different types of mental representation exist and that both can be selectively damaged in representational neglect. The first type is topological [26] and includes mental representations of environments that can be used for navigational purposes. The second type is non-topological [26] and includes mental representations of single or multiple objects that cannot be used for navigational purposes. In our sample, selective representational neglect for topological images was much more frequent (ratio 8∶1) than selective representational neglect for non-topological images. Representational neglect for both topological and non-topological images was less frequent than selective representational neglect for topological images (ratio∶ about 1∶3.4).

The different frequency of the two types of representational neglect may indicate that the sensitivity of the two tests used to assess representational neglect is not the same. Indeed, we cannot exclude that the O'Clock Test, used to assess non-topological representational neglect, might be less sensitive than the topological test. Nevertheless, some of our findings contradict this interpretation. First, the low correlation in the control group between the Familiar Square Description Test and the O'Clock Test indicates that these tests share few cognitive components and thus measure different aspects of mental images. Second, this interpretation does not fit with the observation of double dissociations in patients affected by representational neglect. An alternative interpretation is based on differences in the frame of reference utilized for the images: an egocentric frame of reference has to be used to mentally visualize Piazza del Duomo (Milan) as it appears facing the Duomo, but it may not be necessary for mentally visualizing two clocks. However, we have no proof that an allocentric frame of reference was used in the O'Clock Test. Another possibility is that imagining a public square requires imagining far extrapersonal space, whereas single (even complex) objects can be imagined in both near and far space. Thus, patients with deficits in far extrapersonal space would be unable to describe a familiar square from memory but would be able to imagine two clocks in their reaching space (e.g., on their wrist). On the other hand, patients with deficits in near space would be able to describe a familiar square from memory and imagine two clocks in far space (e.g., two clocks on a far wall or two tower clocks). Thus, it could be that the double dissociation is not limited to the imagery domain, because the standard battery of tests we used to investigate perceptual neglect does not allow assessing deficits in far extrapersonal space.

As hypothesized in previous reports [29] [31] [28] [26], the present data demonstrate the existence of two separate imagery processes that are very likely supported by two independent functional-anatomical neural systems. Trojano and co-workers [50] used the O'Clock Test to explore the neural correlates involved in spatial imagery. Their study showed that mentally comparing pairs of times and judging at which of the two times the clock hands form the widest angle produces prominent cortical activation in the posterior parietal lobes of both hemispheres as well as in the prefrontal cortex. The authors associated this with the attentional demands of different kinds of mental imagery and working memory tasks. Their results provide evidence for the specific convergence of imagery and visual perception pathways in the parietal lobes. We can hypothesize that the mental images generated during the Familiar Square Description Test which are related to previous environmental knowledge might be different and involve cerebral structures generally activated by navigational tasks. For example, in a recent fMRI study Morgan et al. [51] suggested that a map-like representation might exist in the human medial temporal lobe which encodes the coordinates of familiar locations in large-scale, real-world environments. Unfortunately, in the present study we were unable to make a detailed analysis of the neural basis because the patients were submitted to different types of neuroradiological scans (CT, 1-Tesla MRI, 3-Tesla MRI). In any case, the presence of double dissociations in our sample indicates that future studies should attempt to understand whether or not these two mental representations of topological and non-topological images have the same neural networks.

Previous studies reported the scarce prevalence of representational neglect following right-hemisphere lesions [14]. Our results only partially agree with this finding: Representational neglect for non-topological images was quite rare and affected only about 3% of the sample of right brain-damaged patients, whereas the percentage of patients affected by representational neglect for topological images was much higher (i.e., 25%). But when we considered all cases of representational neglect for topological and non-topological images, representational neglect was present in about one-third of the sample, that is, its prevalence was similar to that found for perceptual neglect. Our results show the absence of differences in frequency of occurrence between pure perceptual neglect and pure representational neglect. But a significant difference emerged when we analyzed different types of representational neglect measured by the O'Clock Test and the Familiar Square Description Test. The use of different testing procedures could have led to the discrepancy between the prevalence reported in Bartolomeo et al.'s study [14] and the prevalence found in the present study. In fact, our data suggest that if representational neglect is evaluated by tests requiring the mental representation of non-topological images fewer patients will be diagnosed with the disorder. However, one of the two tests of representational neglect used by Bartolomeo and co-workers [14] was the Familiar Square Description Test, which requires subjects to generate and inspect topological images. Furthermore, the two samples differed along at least two main clinical dimensions: time from onset and lesion site. In fact, Bartolomeo et al. [14] recruited patients in the neurological ward of a general hospital and we recruited patients in a rehabilitation hospital. Usually patients admitted to a rehabilitation hospital have a longer time from onset than patients in a general hospital, who typically are in the acute phase of stroke. In our sample, all but four patients were admitted to the rehabilitation hospital at least 30 days after stroke, 79.17% were admitted two months after stroke and 15% more than a year after stroke. Furthermore, only stroke patients with motor impairments are admitted to rehabilitation hospitals, whereas all kinds of stroke patients are admitted to neurological wards. Therefore, most of the patients admitted to rehabilitation hospitals have rather large lesions resulting from damage to the middle cerebral artery, involving the motor network. Instead, all patients suffering from stroke (including those with small lesions), those with or without transient motor impairments and those with lesions in different cerebral territories are admitted to neurological wards of general hospitals.

As mentioned above, in the present study it was impossible to localize the neural basis of ReprNeg for topological and non-topological images by analyzing structural neuroimages. Furthermore, different variables prevented us from drawing any conclusions about the different anatomical basis of the two ReprNeg disorders. First, the two groups of ReprNeg (i.e. T-ReprNeg and Nt-ReprNeg) were numerically dishomogeneous and very few patients showed selective Nt-ReprNeg. Second, most patients had large, mostly overlapping lesions in the territory of the middle cerebral artery. The large amount of overlapping undermines the hypothesis that ReprNeg derives principally from damage to specific areas involved in processing topological and non-topological images. It is possible that the different occurrence of the two types of representational neglect was due to the different occurrence of lesions disconnecting the posterior parietal areas (more active in processing non-topological images) or medial temporal lobe posterior areas (more active in processing environmental representation) from the anterior areas involved in visuo-spatial working memory, a cognitive process used in processing mental images. Instead, we can speculate that the co-occurrence of representational neglect for both topological and non-topological images is due to damage in both networks that process topological and non-topological material or to a posterior callosal disconnection (as in the patient described by Rode and colleagues) [52], which would impede the left hemisphere from receiving adequate input from the right hemisphere and result in defective exploration of the left part of any kind of generated image.

These hypotheses are still speculative and are difficult to test using standard neuro-radiological scans (such as the ones we used). In fact, specific neuro-radiological studies are needed to show the connectivity between different brain regions (i.e., Diffusion Tensor Imaging).

The present data highlight the importance of assessing representational neglect in right brain-damaged patients, because, despite its frequency, this disorder often goes undiagnosed. Studies analyzing the consequences of representational neglect in everyday life and functional recovery after brain damage are also needed. We believe that identifying different forms of representational neglect is of great clinical importance to avoid the discovery of navigational disorders when patients are about to be discharged from hospital to return to their daily life activities. Indeed, this scenario has negative effects on patients' general mood and autonomy. Early detection of different forms of representational neglect can help clinicians plan goal-directed rehabilitative programs that go beyond visuo-explorative training and also treat navigational disorders.

## References

[1] Bisiach E, Vallar G (2000) Unilateral neglect in humans. In FBoller and JGrafman (Eds.). Handbook of Neuropsychology. Amsterdam: Elsevier, pp. 459–502.

[2] Heilman KM, Valenstein E, Watson RT (2000) Neglect and related disorders. Semin Neurol 20: 463–470. 11149702 10.1055/s-2000-13179

[3] Kerkhoff G (2001) Spatial hemineglect in humans. Progress in Neurobiology 63: 1–27. 11040416 10.1016/s0301-0082(00)00028-9

[4] Weintraub S, Mesulam M-M (1988) Visual hemispatial inattention: stimulus parameters and exploratory strategies. J Neurol Neurosurg Psychiatry 51: 1481–8. 3221214 10.1136/jnnp.51.12.1481 PMC1032760

[5] Vallar G (2001) Extrapersonal visual unilateral spatial neglect and its neuroanatomy. Neuroimage 14: S52–58. 11373133 10.1006/nimg.2001.0822

[6] Vallar G, Bottini G, Paulesu E (2003) Neglect syndromes: the role of the parietal cortex. In: Siegel AM, Andersen RA, Freund H-J, Spencer DD, (eds.) Advances in neurology. The parietal lobes. Philadelphia: Lippincott Williams & Wilkins, pp. 293–319. 12894416

[7] Bisiach E, Perani D, Vallar G, Berti A (1986) Unilateral neglect: Personal and extrapersonal. Neuropsychologia 24: 759–767. 3100983 10.1016/0028-3932(86)90075-8

[8] Guariglia C, Antonucci G (1992) Personal and extrapersonal space: A case of neglect dissociation. Neuropsychologia 30: 1001–1009. 1470335 10.1016/0028-3932(92)90051-m

[9] Guariglia C, Padovani A, Pantano P, Pizzamiglio L (1993) Unilateral neglect restricted to visual imagery. Nature 364: 235–237. 8321319 10.1038/364235a0

[10] Pizzamiglio L, Guariglia C, Nico D, Padovani A (1996) Two separate systems for space cognition. In J MLevelt (Ed.), Advanced psycholinguistics (pp. 157–162). Nijmegen, The Netherlands: Max Plank Institute.

[11] Anderson B (1993) Spared awareness for the left side of internal visual images in patients with left-sided extrapersonal neglect. Neurology 43: 213–216. 8423890 10.1212/wnl.43.1_part_1.213

[12] Beschin N, Basso A, Della Sala S (2000) Perceiving left and imagining right: dissociation in neglect. Cortex 36: 401–414. 10921667 10.1016/s0010-9452(08)70849-9

[13] Bisiach E, Luzzatti C (1978) Unilateral neglect of representational space. Cortex 14: 129–133. 16295118 10.1016/s0010-9452(78)80016-1

[14] Bartolomeo P, D'Erme P, Gainotti G (1994) The relationship between visuospatial and representational neglect. Neurology 44: 1710–1714. 7936302 10.1212/wnl.44.9.1710

[15] Bartolomeo P, Bachoud-Levi AC, Azouvi P, Chokron S (2005) Time to imagine space: a chronometric exploration of representational neglect. Neuropsychologia 43: 1249–1257. 15949509 10.1016/j.neuropsychologia.2004.12.013

[16] Bisiach E (1993) Mental representation in unilateral neglect and related disorders. Quarterly Journal of Experimental Psychology A 46: 435–461. 10.1080/14640749308401056 8378550

[17] Coslett HB (1997) Neglect in vision and visual imagery: a double dissociation. Brain 120: 1163–1171. 9236629 10.1093/brain/120.7.1163

[18] Beschin N, Cocchini G, Della Sala S, Logie RH (1997) What the eyes perceive, the brain ignores: a case of pure unilateral representational neglect. Cortex 33: 3–26. 9088719 10.1016/s0010-9452(97)80002-0

[19] Logie RH, Della Sala S, Beschin N, Denis M (2005) Dissociating mental transformations and visuo-spatial storage in working memory: evidence from representational neglect. Memory 13: 430–434. 15948629 10.1080/09658210344000431

[20] Grossi D, Modafferi A, Pelosi L, Trojano L (1989) On the different roles of the cerebral hemispheres in mental imagery: the “O'Clock Test” in two clinical cases. Brain and Cognition 10: 18–27. 2713142 10.1016/0278-2626(89)90072-9

[21] Ortigue S, Viaud-Delmon I, Michel CM, Blanke O, Annoni JM, et al (2003) Pure representational neglect for far space. Neurology 60: 2000–2002.12821753

[22] Arduino LS, Marinelli CV, Pasotti F, Ferrè ER, Bottini G (2012) Representational neglect for words as revealed by bisection tasks. Journal of Neuropsychology 6: 43–64. 22257574 10.1111/j.1748-6653.2011.02003.x

[23] Kosslyn SM (1980) Image and mind. Cambridge, MA: Harvard University Press.

[24] Bisiach E, Luzzatti C, Perani D (1979) Unilateral neglect, representational schema and consciousness. Brain 102: 609–618. 497807 10.1093/brain/102.3.609

[25] Ogden JA (1985) Contralesional neglect of constructed visual images in right and left brain damaged patients. Neuropsychologia 23: 273–277. 4000463 10.1016/0028-3932(85)90112-5

[26] Guariglia C, Pizzamiglio L (2006) Spatial navigation: cognitive and neuropsychological aspects. In TVecchi, & GBottini (Eds.), Imagery And Spatial Cognition: Methods, Model and Cognitive Assessment (pp. 285–297). Amsterdam/Philadelphia: John Benjamins Publishing Company.

[27] Guariglia C, Piccardi L, Iaria G, Nico D, Pizzamiglio L (2005) Representational neglect and navigation in real space. Neuropsychologia 43: 1138–1143. 15817171 10.1016/j.neuropsychologia.2004.11.021

[28] Piccardi L, Bianchini F, Zompanti L, Guariglia C (2008) Pure representational neglect and navigational deficits in a case with preserved visuo-spatial working memory. Neurocase 14: 329–342. 18792838 10.1080/13554790802366012

[29] Palermo L, Nori R, Piccardi L, Giusberti F, Guariglia C (2010a) Environmental and object mental images in patients with representational neglect: two cases reports. Journal of the International Neuropsychological Society 16: 921–932. 20331913 10.1017/S1355617710000305

[30] Palermo L, Ranieri G, Nemmi F, Guariglia C (2012) Cognitive maps in imagery neglect. Neuropsychologia 50: 904–12. 22310104 10.1016/j.neuropsychologia.2012.01.030

[31] Palermo L, Piccardi L, Nori R, Giusberti F, Guariglia C (2010b) Does hemineglect affect visual mental imagery? Imagery deficits in representational and perceptual neglect. Cognitive Neuropsychology 27: 115–133. 20721762 10.1080/02643294.2010.503478

[32] Folstein MF, Folstein SE, McHugh PR (1975) “Mini-mental state”. A practical method for grading the cognitive state of patients for the clinician. Journal of Psychiatric Research 12: 189–198. 1202204 10.1016/0022-3956(75)90026-6

[33] Spinnler H, Tognoni G (1987) Standardizzazione e taratura italiana di test neuropsicologici [Standardization and validation of neuropsychological Italian tests]. Italian Journal of Neurological Sciences 8 (suppl.) 1–120. 3330072

[34] Ciurli P, Marangolo P, Basso A (1996) Esame del Linguaggio II [Examination of Language II]. O.S. Organizzazioni Speciali. Florence, Italy.

[35] Raven JC (1938) Standard Progressive Matrices: Sets A, B, C, D and EHK. London: Lewis.

[36] Street RF (1931) A Gestalt completion test. Teachers College Record 33: 280–282.

[37] Pizzamiglio L, Judica A, Razzano C, Zoccolotti P (1989) Toward a comprehensive diagnosis of visual spatial disorders in unilateral brain damaged patients. Psychological Assessment 5: 199–218.

[38] Diller L, Weinberg J (1977) Hemi-inattention and hemisphere specialization. In EAWeinstein & RPFriedland (Eds.), Advances in neurology (Vol. 18, pp. 63–82). New York: Raven Press.

[39] Pizzamiglio L, Fasotti L, Jehkonen M, Antonucci G, Magnotti L, et al (2004) The use of optokinetic stimulation in rehabilitation of the hemineglect disorder. Cortex 40: 441–50. 15259325 10.1016/s0010-9452(08)70138-2

[40] Albert ML (1973) A simple test of visual neglect. Neurology 23: 658–664. 4736313 10.1212/wnl.23.6.658

[41] Massironi M, Antonucci G, Pizzamiglio L, Vitale MV, Zoccolotti P (1988) The Wundt–Jastrow illusion in the study of spatial hemi-inattention. Neuropsychologia 26: 1661–1666. 10.1016/0028-3932(88)90039-5 3242500

[42] Paivio A (1978) Comparison of mental clock. J Exp Psychol (Hum Percept) 4: 61–71. 627851 10.1037//0096-1523.4.1.61

[43] Trojano L, Grossi D (1994) A critical review of mental imagery defects. Brain Cognition 24: 213–243. 8185895 10.1006/brcg.1994.1012

[44] Crawford JR, Garthwaite PH (2005) Evaluation of criteria for classical dissociations in single-case studies by Monte Carlo simulation. Neuropsychology 19: 664–678. 16187885 10.1037/0894-4105.19.5.664

[45] Crawford JR, Howell DC (1998) Comparing an individual's test score against norms derived from small samples. Clinical Neuropsychology 12: 482–486.

[46] Wojciulik E, Husain M, Clarke K, Driver J (2001) Spatial working memory deficit in unilateral neglect. Neuropsychology 39: 390–396. 10.1016/s0028-3932(00)00131-7 11164877

[47] Della Sala S, Logie RH, Beschin N, Denis M (2004) Preserved visuo-spatial transformations in representational neglect. Neuropsychologia 42: 1358–1364. 15193943 10.1016/j.neuropsychologia.2004.02.011

[48] Cristinzio C, Bourlon C, Pradat-Diehl P, Trojano L, Grossi D, et al (2009) Representational neglect in “invisible” drawing from memory. Cortex 45: 313–7. 18718580 10.1016/j.cortex.2008.03.013

[49] Corsi PM (1972) Human memory and the medial temporal region of the brain. Dissertation Abstract International 34 (02) 891 B (University micro-lms No. AA105-77717).

[50] Trojano L, Grossi D, Linden DEJ, Formisano E, Hacker H, et al (2000) Matching two imagined clocks: the functional anatomy of spatial analysis in the absence of visual stimulation. Cerebral Cortex 10: 473–481. 10847597 10.1093/cercor/10.5.473

[51] Morgan LK, MacEvoy SP, Aguirre GK, Epstein RA (2011) Distances between Real-World Locations are represented in the human hippocampus. The Journal of Neuroscience 31: 1238–1245. 21273408 10.1523/JNEUROSCI.4667-10.2011 PMC3074276

[52] Rode G, Cotton F, Revol P, Jacquin-Courtois S, Rossetti Y, et al (2010) Representation and disconnection in imaginal neglect. Neuropsychologia 48: 2903–11. 20621588 10.1016/j.neuropsychologia.2010.05.032

